# The risk factor of clinical relapse in ulcerative colitis patients with low dose 5-aminosalicylic acid as maintenance therapy: A report from the IBD registry

**DOI:** 10.1371/journal.pone.0187737

**Published:** 2017-11-06

**Authors:** Tomohiro Fukuda, Makoto Naganuma, Shinya Sugimoto, Kosaku Nanki, Shinta Mizuno, Makoto Mutaguchi, Yoshihiro Nakazato, Nagamu Inoue, Haruhiko Ogata, Yasushi Iwao, Takanori Kanai

**Affiliations:** 1 Division of Gastroenterology and Hepatology, Department of Internal Medicine, Keio University School of Medicine, Tokyo, Japan; 2 Center for Diagnostic and Therapeutic Endoscopy, Keio University School of Medicine, Tokyo, Japan; 3 Center for Preventive Medicine, Keio University School of Medicine, Tokyo, Japan; Kurume University School of Medicine, JAPAN

## Abstract

**Background:**

5-Aminosalicylic acids (5-ASA) are effective for ulcerative colitis (UC) as a maintenance therapy. It is not clear when and how to reduce a dose of 5-ASA after inducing remission. We aimed to investigate the clinical characteristics and evaluate the risk factors of relapse for UC patients receiving 5-ASA.

**Methods:**

The medical records of prospectively registered UC patients who received oral 5-ASA as maintenance therapy between January and December 31, 2014, were investigated. The patients’ clinical characteristics in a 2-year follow-up were compared between a relapse group and a remission group.

**Results:**

Of 527 UC patients receiving only oral 5-ASA, 390 (74.0%) maintained remission and 137 (26.0%) relapsed during the follow-up period. Multivariable analysis indicated that a shorter duration of disease remission (*p* < 0.001, OR: 1.24, 95% CI: 1.12–1.38) was statistically significant for each comparison between the remission and relapse groups among all the patients. Risk factors for clinical relapse were a shorter duration of disease remission (*p* <0.001, OR: 1.17, 95% CI: 1.04–1.33) in the high-dose 5-ASA group and a shorter duration of disease remission (*p* = 0.003, OR: 1.45, 95% CI: 1.13–1.89) and a history of steroid use (*p* = 0.048, OR: 4.73, 95% CI: 1.01–22.2) in the low-dose group.

**Conclusions:**

A dose reduction of 5-ASA might be cautiously selected in UC patients with a history of steroid use and a shorter duration of disease remission.

## Introduction

Ulcerative colitis (UC) is a chronic idiopathic inflammatory disease of the large intestine that is characterized by periods of remission and relapse [1]. 5-Aminosalicylic acids (5-ASA) are the first-line drug and an effective treatment for induction in patients with mild to moderate UC [2]. Once remission is achieved with any therapeutic agents, up to 70% of patients not receiving maintenance treatment are expected to relapse within a year [3].

The effect of 5-ASA on the maintenance of remission was confirmed in a Cochrane systematic review [4]. The results of a recent meta-analysis indicated that the odds ratio for failure to maintain clinical or endoscopic remission for 5-ASA versus the placebo was 0.47 [5]. A recent study in children newly diagnosed with UC found that 40% of the children started on 5-ASA as the primary maintenance therapy at diagnosis were in corticosteroid-free remission after 1 year of treatment [6].

Although several studies have reported the usefulness for inducing and maintaining remission, there is still little evidence that 5-ASA significantly prevents relapse dose-dependently. Paoluzi *et al*. observed that 2.4 g daily of oral 5-ASA seems to be better at preventing and delaying relapses of UC than 1.2 g daily [7]. Fockens *et al*. reported that patients in a group that received 3.0 g showed a lower relapse rate at 1 year than a 1.5 g dose group [8]. Rubin *et al*. found that long-term higher-dose 5-ASA treatments probably prevented clinical relapse more effectively than a lower dose in UC patients without complete remission [9]. These results suggest that treatments with higher-dose 5-ASA may contribute to a better clinical outcome. However, other studies have shown that the efficacy of preventing relapse was not significantly different in patients even taking more than 2.5 g of 5-ASA daily. Furthermore, the efficacy for maintenance of clinical remission was comparable between patients taking a high dose of 5-ASA and those taking a low dose when treatment adherence was moderate to excellent [10]. These results indicated that it may be difficult to decide when to reduce the dose of 5-ASA after clinical remission has been induced. In addition, some patients want to receive a lower dose of 5-ASA. However, no definitive criteria for dose reduction of 5-ASA in maintenance therapy have been established. In this study, we investigated the clinical characteristics and risk factors of relapse for UC patients who received 5-ASA as maintenance therapy, especially those who received a low dose of 5-ASA.

## Materials and methods

### Study design and patients

We conducted a review of the medical records of UC patients who visited an outpatient clinic of the Division of Gastroenterology and Hepatology, Department of Internal Medicine, Keio University Hospital (Tokyo, Japan), between January 1, 2014, and December 31, 2014, from our database of prospectively registered UC patients and who could be evaluated in detail based on their clinical information. We performed detailed analyses of 1325 UC patients who were treated with oral 5-ASA therapy. Three formulations of 5-ASA were identified: Asacol^®^, Pentasa^®^, and Salazosulfapyridine^®^. We excluded patients who received combination therapy for UC (thiopurine, corticosteroid, tacrolimus, cyclosporine, infliximab, adalimumab, and granulocyte apheresis).

### Ethical considerations

This study was approved by the Ethics Committee of Keio University School of Medicine (no. 20150210) and was conducted in accordance with the principles of the Declaration of Helsinki. Because no informed consent was required for this retrospective observational study, we obtained the patient’s consent to participate by posting information about this study in Keio University Hospital. Patient records and information were anonymized and de-identified before analysis.

### Study procedures

The patients’ demographic data and disease characteristics, including their gender, age, age at onset of symptoms, duration of disease, duration of disease remission, site of the disease, serum albumin (Alb) level, total cholesterol (TC) level, white blood cell (WBC) count, hemoglobin (Hb), platelet (Plt) level, serum C-reactive protein (CRP) level, erythrocyte sedimentation rate (ESR), endoscopic Mayo score (MES), history of steroid use, and history of thioprine use, were collected. We followed the patients’ progress for 2 years from their first visit between January 1, 2014, and December 31, 2014, and classified them into 2 groups as follows: a remission group (partial Mayo (pMayo) score of ≤ 1 for 2 years) and a relapse group (pMayo score of ≥ 3 at least once in 2 years or received new treatment for clinical symptoms of UC). It is difficult to classify pMayo score of 2 into relapse group or remission group. For this reason, patients with pMayo score of 2 at least once during observation period were excluded in this study. PMayo scores were assessed and recorded by physicians at the original time of each visit. Subsequently, patients were subclassified into a high-dose group (HDG) and a low-dose group (LDG) according to the daily dose of 5-ASA. HDG consisted of patients who received at least 3.6 g daily of Asacol^®^, over 3.0 g daily of Pentasa^®^, or over 3.0 g daily of Salazosulfapyridine^®^, and LDG was consisted of others. The patients in HDG and LDG were additionally subclassified into remission groups and relapse groups. In the manner described above, we analysed the patients’ demographic data and disease characteristics between the relapse and remission groups. Information regarding adherence with 5-ASA was also collected. Poor adherence was defined as the presence of one of the following: (i) declined prescriptions two consecutive times in an outpatient clinic, (ii) poor adherence noted in medical records, (iii) self-determined dose reduction or discontinuation of taking medicine, and (iv) hospital visit more than 2 months after the last regular visit.

### Statistical analysis

Paired variables were evaluated by Fischer’s exact test or Student’s t-test, and categorical variables were compared using the chi-squared or Kruskal-Wallis tests. Logistic regression was used to perform a multivariate analysis of risk factors for disease relapse, gender, age, age at onset of symptoms, duration of disease, duration of disease remission, MES, dose of 5-ASA (HDG or LDG), history of steroid use, and history of thiopurine use. All of the statistical analyses were performed using IBM SPSS Statistics version 23 (IBM Corp., Armonk, N.Y., USA). Two-sided *p* values were considered statistically significant at a level of <0.05.

## Results

### Patient profile

**Fig 1:**
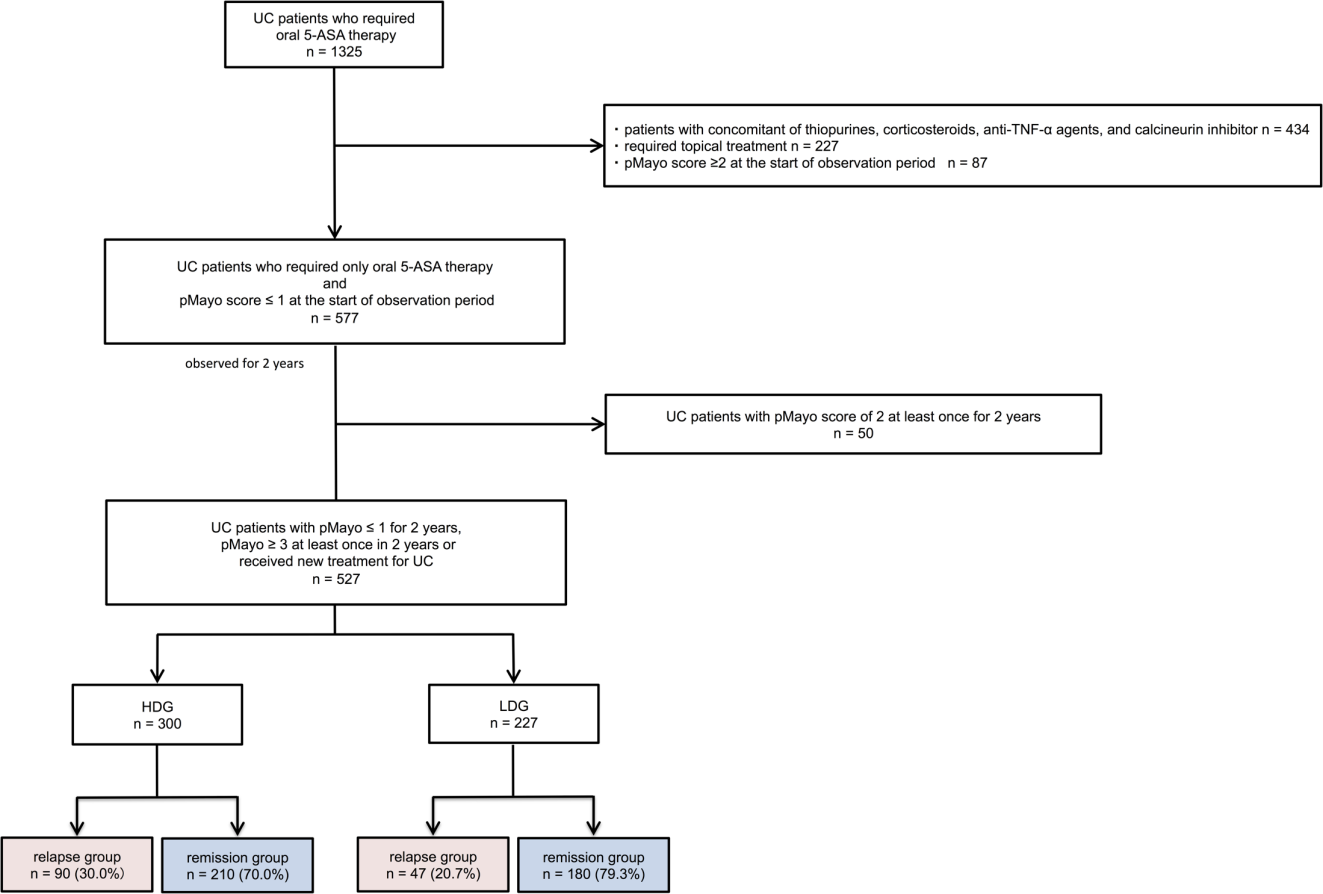
Flowchart of participants in this study. A total of 1325 UC patients with oral 5-ASA maintenance therapy were identified. We excluded 661 patients who received thiopurines (6-mercaptopurine and azathioprine), anti-TNF-α agents (adalimumab and Infliximab), tacrolimus, and topical treatment such as a suppository or enema, and 87 patients who had a pMayo score of ≥ 2 at the start of the observation period. Among 577 patients, 50 patients with pMayo score of 2 at least once during observation period were excluded. Consequently, 527 patients were included in this study. Of these, 300 received a high dose of 5-ASA and 227 received a low dose. Ninety patients (30.0%) in HDG and 47 patients (20.7%) in LDG relapsed. The difference in relapse rates between HDG and LDG was statistically significant (*p* = 0.016).

Between January 1, 2014, and December 31, 2014, 1325 patients were treated with oral 5-ASA to maintain clinical remission of UC. Patients with concomitant use of thiopurines (6-mercaptopurine and azathioprine), anti-TNF-α agents (adalimumab and infliximab), and tacrolimus were excluded from this study and 891 UC patients were identified. In our institution, VSL-3, methotrexate and vedolizumab are never used (these agents have not yet been approved in Japan). Of the 891 patients, 227 who received topical treatment, such as a suppository or enema, and 87 patients with a pMayo score of ≥ 2 at the start of observation period were excluded. as a result, a total of 577 patients with a pMayo score of ≤ 1 who were treated with oral 5-ASA alone were identified. Among 577 patients, 50 patients with pMayo score of 2 at least once during observation period were excluded. Consequently, 527 patients were included in this study. Of these, 300 received a high dose of 5-ASA and 227 received a low dose (Fig 1). The baseline characteristics of the 527 patients are shown in Table 1. The mean duration of disease and the duration of disease remission were 14.8 and 4.16 years, respectively. The mean MES was 0.68 (N = 241). Ultimately, 300 and 227 patients were classified into HDG and LDG, respectively (Fig 1). Table 1. showed baseline characteristics of HDG and LDG. Expectedly, there were statistically significant in shorter duration of disease remission, extent of disease, MES, history of steroid use, history of thiopurine use, and clinical relapse between 2 groups.

**Table 1:** Baseline characteristics of the 527 patients in this study.

	total n = 527	HDG n = 300	LDG n = 227	*p* value
Male; n (%)	282 (53.5)	163(54.3)	119(52.4)	0.72
Age, years; mean (SD)	47.99 (14.6)	47.3 (14.6)	48.4(14.6)	0.40
Age at onset, years; mean (SD)	33.3 (13.2)	32.7(13.1)	34.0(13.4)	0.30
Duration of disease, years; mean (SD)	14.8 (9.45)	14.7 (9.60)	14.9 (9.29)	0.79
Duration of disease remission, years; mean (SD)	4.16 (4.59)	3.19 (3.84)	5.44(5.18)	<0.001
Extent of disease				
Extensive; n (%)	196 (37.2)	130(43.3)	66(29.1)	<0.001
Alb level, g/dl; mean (SD)	4.33 (0.33)	4.32 (0.34)	4.35 (0.32)	0.32
TC level, mg/dl; mean (SD)	195.6 (34.2)	195.2 (33.4)	196.1 (35.5)	0.80
WBC counts, 10^3^/μl; mean (SD)	5.93 (1.72)	5.86 (1.75)	6.01 (1.68)	0.39
Hb, g/dl; mean (SD)	13.6 (1.75)	13.5 (1.79)	13.7 (1.69)	0.19
Plt level, 10^4^/μl; mean (SD)	25.9 (6.34)	26.4 (6.47)	25.3 (6.13)	0.080
CRP level, mg/dl; mean (SD)	0.16 (0.60)	0.18 (0.76)	0.13 (0.25)	0.38
ESR level, mm/hr; mean (SD)	9.47 (9.50)	9.64(9.83)	9.21(9.02)	0.72
Mayo endoscopic score; mean (SD)	0.68(0.73)	0.80(0.70)	0.49(0.79)	0.001
Medical history				
Prednisolone (+); n (%)	128 (24.8)	89 (29.7)	39 (17.2)	0.001
Thiopurine (+); n (%)	20(3.8)	16(5.3)	4(1.8)	0.038
Clinical relapse (+); n(%)	137 (26.0)	90(30.0)	47 (20.7)	0.016

n, number; SD, standard deviation; HDG, high dose group; LDG, low dose group; Alb, serum albumin; TC, total cholesterol; WBC, white blood cell count; Hb, haemoglobin; Plt, platelet; CRP serum C-reactive protein; ESR, erythrocyte sedimentation rate.

### Risk factors for clinical relapse in all cohorts

**Fig 2:**
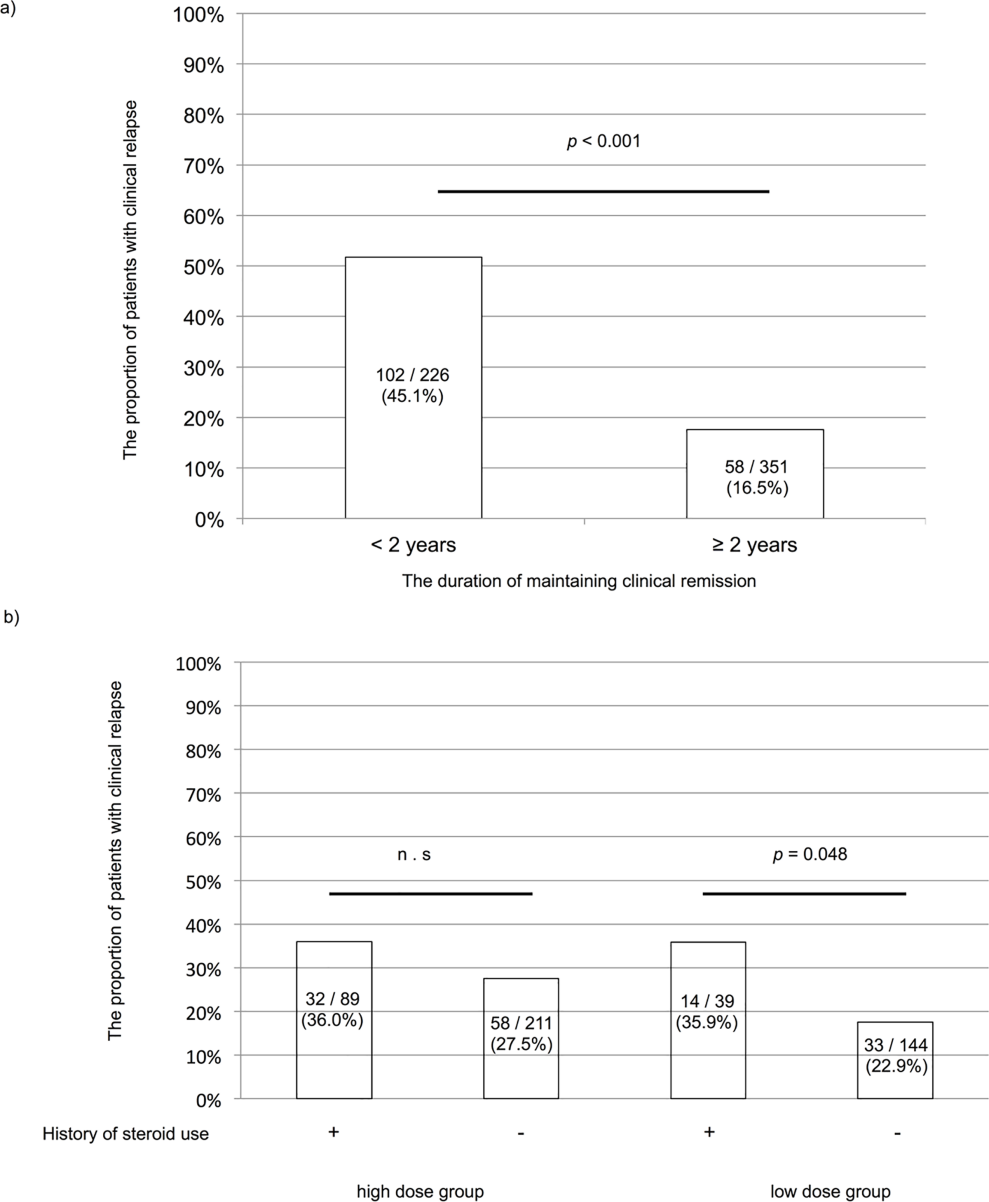
Relationship between the dose of 5-ASA and relapse in participants. (a) The proportion of patients with clinical relapse was compared between patients with <2 and those with ≥2 years of disease remission. (b) The proportion of patients with clinical relapse was statistically significant higher in low dose group with a history of steroid use than that without a history of steroid use. However, there was no statistically significant difference regardless of the previous steroid use among high dose group.

Of the 527 patients, 390 (64.0%) maintained remission and 137 (26.0%) relapsed during the 2-years observational period. Additional treatment for UC in the relapse group was mainly topical treatment (59 patients), corticosteroids (22 patients), and an increased 5-ASA dose (22 patients). Among them, mean dosing period of 5-ASA were 175.6 months (HDG 175.0 months, LDG 176.5 months). As shown in Table 2, age (*p* = 0.031), duration of disease remission (*p* < 0.001), serum albumin level (*p* = 0.043), 5-ASA dose (*p* = 0.016), gender (*p* = 0.04) and history of steroid use (*p* = 0.03) were statistically significant for each comparison between the remission and relapse groups. Multivariate analysis indicated that earlier age at onset (*p* = 0.023, OR: 1.03, 95% CI: 1.004–1.06), shorter duration of disease remission (*p* < 0.001, OR: 1.24, 95% CI: 1.12–1.38) and MES (*p* = 0.021, OR: 1.63, 95% CI: 1.08–2.47) were risk factors for clinical relapse. Dosage of 5-ASA was not statistically significant in multivariable analysis (p = 0.065). As shown in Fig 2A, there were statistically significant results for each comparison between <2 and ≥2 years of disease remission (*p* < 0.001). The relapse rate was 45.1% among the patients with <2 years of remission and 16.5% among those with ≥2 years of remission.

**Table 2:** Risk factors for disease relapse in all 527 patients.

			Univariate analyses			Multivariate analyses		
	Remission (N = 390)	Relapse (N = 137)	OR	95% CI	*p* value	OR	95% CI	*p* value
Age, years; mean	48.7	45.5	-	0.28–5.97	0.031			
Age at onset, years; mean	33.9	31.5	-	-0.14–5.00	0.064	1.03	1.004–1.05	0.023
Duration of disease, years; mean	15.0	14.1	-	-0.89–2.78	0.31			
Duration of disease remission, years; mean	4.8	2.4	-	1.57–3.32	<0.001	1.24	1.11–1.38	<0.001
serum Alb, g/dl; mean	4.4	4.3	-	0.003–0.19	0.043			
TC level, mg/dl; mean	197	191	-	-1.63–14.0	0.12			
WBC counts, 10^3^/μl; mean	5.9	6.1	-	-0.70–0.13	0.18			
Hb, g/dl; mean	13.6	13.4	-	-0.15–0.61	0.24			
Plt level, 10^4^/μl; mean	256	266	-	-23.6–3.99	0.16			
CRP level, mg/dl; mean	0.17	0.15	-	-0.11–0.15	0.73			
ESR level, mm/h; mean	9.0	10	-	-4.90–1.48	0.29			
Mayo endoscopic score; mean	0.60	0.89	-	-0.51–-0.66	0.012	1.63	1.08–2.47	0.021
HDG, n	210	90	1.64	1.10–2.46	0.016			
LDG, n	180	47						
Male, n	219	63	1.50	1.02–2.22	0.04			
Female, n	171	74						
Previous steroid use (+), n	82	46	1.90	1.24–2.92	0.03			
Previous steroid use (-), n	308	91						
Previous thiopurine use (+), n	18	7	1.11	0.45–2.72	0.82			
Previous thiopurine use (-), n	372	130						

n, number; SD, standard deviation; HDG, high dose group; LDG, low dose group; Alb, serum albumin; TC, total cholesterol; WBC, white blood cell count; Hb, haemoglobin; Plt, platelet; CRP serum C-reactive protein; ESR, erythrocyte sedimentation rate.

In the assessment of the relationship between poor adherence and relapse, 73 patients’ adherence was poor, and 49 of those patients relapsed (67.1%). Relapse rates were 100% (35/35) in HDG and 48.7% (13/38) in LDG. Poor adherence was not statistically significant between the remission and relapse groups in 300 HDG patients (*p* = 0.84). However, poor adherence increased the risk of relapse in LDG patients (*p* = 0.03).

### Risk factors for clinical relapse in HDG

The difference in the relapse rate between HDG and LDG patients was statistically significant. Therefore we next analyzed the risk factors of HDG and LDG patients separately as clinical background was different between HDG and LDG patients.

The numbers of patients in the remission and relapse groups are shown in Fig 1. Of the 300 HDG patients, 210 (70.0%) remained in remission and 90 (30.0%) relapsed. As shown in Table 3, univariate analysis indicated that duration of disease remission (*p* < 0.001) and MES (*p* = 0.041) were risk factors for clinical relapse. Multivariate analysis indicated that each comparison between the remission and relapse groups in HDG revealed that a shorter duration of disease remission (*p* <0.001, OR: 1.17, 95% CI: 1.04–1.33) and MES (*p* = 0.047, OR: 1.62, 95% CI: 1.01–2.62) correlated with relapse.

**Table 3:** Risk factors for disease relapse in HDG.

			Univariate analyses			Multivariate analyses		
	Remission (N = 210)	Relapse (N = 90)	OR	95% CI	*p* value	OR	95% CI	*p* value
Age, years; mean	48.2	45.5	-	-0.79–6.10	0.13			
Age at onset, years; mean	33.1	31.8	-	-1.73–4.48	0.38			
Duration of disease, years; mean	15.1	13.7	-	-0.85–3.69	0.22			
Duration of disease remission, years; mean	3.8	1.9	-	0.96–2.82	<0.001	1.17	1.04–1.33	<0.001
serum Alb, g/dl; mean	4.3	4.3	-	-0.66–0.119	0.57			
TC level, mg/dl; mean	198	190	-	-2.10–16.5	0.13			
WBC counts, 10^3^/μl; mean	5.8	6.0	-	-0.71–0.24	0.33			
Hb, g/dl; mean	13.5	13.4	-	-0.45–0.52	0.87			
Plt level, 10^4^/μl; mean	263	265	-	-20.9–16.0	0.79			
CRP level, mg/dl; mean	0.20	0.16	-	-0.15–0.23	067			
ESR level, mm/h; mean	9.0	11.1	-	-6.04–1.69	0.27			
Mayo endoscopic score; mean	0.71	1.00	-	-0.56–0.017	0.041	1.62	1.005–2.62	0.047
Male, n	115	48	1.05	0.65–1.73	0.82			
Female, n	95	42						
Previous steroid use (+), n	57	32	1.48	0.87–2.51	0.14			
Previous steroid use (-), n	153	58						
Previous thiopurine use (+), n	11	6	1.06	0.36–3.16	0.91			
Previous thiopurine use (-), n	169	70						

n, number; SD, standard deviation; HDG, high dose group; LDG, low dose group; Alb, serum albumin; TC, total cholesterol; WBC, white blood cell count; Hb, haemoglobin; Plt, platelet; CRP serum C-reactive protein; ESR, erythrocyte sedimentation rate.

### Risk factors for clinical relapse in LDG

Of the 227 LDG patients, 180 patients (79.3%) maintained remission and 47 (20.7%) relapsed (Fig 1). As shown in Table 4, age at onset (*p* = 0.025), duration of disease remission (*p* < 0.001), gender (*p* = 0.002), history of steroid use (*p* = 0.001), and poor adherence (p = 0.03) were statistically significant for each comparison between the remission and relapse groups in LDG. Multivariate analysis indicated that shorter duration of disease remission (*p* = 0.003, OR: 1.45, 95% CI: 1.13–1.89), female (p = 0.019, OR: 4.31, 95% CI 1.26–14.7), and the history of steroid use (*p* = 0.048, OR: 4.73, 95% CI: 1.01–22.2) were risk factors for clinical relapse. Fig 2B showed relationship between history of steroid use and dosage of 5-ASA. Although there were not statistically significant whether patients had history of steroid use or not in HDG, patients with history of steroid use had higher relapse risk in LDG.

**Table 4:** Risk factors for disease relapse in LDG.

			Univariate analyses			Multivariate analyses		
	Remission (N = 180)	Relapse (N = 47)	OR	95% CI	*p* value	OR	95% CI	*p* value
Age, years; mean	49.2	45.5	-	-0.14–8.36	0.13			
Age at onset, years; mean	34.8	30.8	-	0.17–8.12	0.025			
Duration of disease, years; mean	15.0	14.7	-	-2.75–3.28	0.86			
Duration of disease remission, years; mean	3.27	6.01	-	1.09–4.37	0.001	1.45	1.13–1.89	0.003
serum Alb, g/dl; mean	4.4	4.3	-	-0.012–0.27	0.072			
TC level, mg/dl; mean	196	193	-	-12.6–20.4	0.64			
WBC counts, 10^3^/μl; mean	5.9	6.4	-	-1.21–0.21	0.16			
Hb, g/dl; mean	13.8	13.3	-	-0.027–1.06	0.062			
Plt level, 10^4^/μl; mean	24.9	26.9	-	-42.3–2.92	0.086			
CRP level, mg/dl; mean	0.13	0.12	-	-0.079–0.11	0.74			
ESR level, mm/h; mean	9.1	9.6	-	-6.52–5.64	0.88			
Mayo endoscopic score; mean	0.46	0.63	-	-0.56–0.22	0.373			
Male, n	104	15	2.92	1.48–5.77	0.002	4.31	1.26–14.7	0.019
Female, n	76	32						
Previous steroid use (+), n	25	14	2.63	1.24–5.59	0.001	4.73	1.01–22.2	0.048
Previous steroid use (-), n	155	33						
Previous thiopurine use (+), n	4	0	-	-	0.30			
Previous thiopurine use (-), n	176	47						

n, number; SD, standard deviation; HDG, high dose group; LDG, low dose group; Alb, serum albumin; TC, total cholesterol; WBC, white blood cell count; Hb, haemoglobin; Plt, platelet; CRP serum C-reactive protein; ESR, erythrocyte sedimentation rate

## Discussion

5-ASA is effective in both inducing and maintaining remission in patients with mild to moderate UC. Although meta-analysis has shown the usefulness of maintenance in 5-ASA therapy in UC patients [4, 5], the effective dose of 5-ASA has not been well investigated. The Toronto consensus guideline indicated that at least 2 g of oral 5-ASA should be continued to maintain complete remission in patients with oral 5-ASA-induced complete remission of active UC [11]. However, it is not clear whether a higher-dose of 5-ASA is required for a long duration to maintain clinical remission. In addition, risk factors of relapse for UC have not been investigated, particularly in patients who receive a lower dose of 5-ASA. In the present study, we demonstrated that a shorter duration of disease remission, history of steroid use and gender were associated with a higher risk of relapse in patients with LDG. From the results of several studies [12], there are controversies concerning the gender. Therefore, we have thought that gender was not usefully predictive factor of relapse disease. Our study highlights the importance of the duration of disease remission and history of steroid use when considering whether to reduce the dose of 5-ASA.

In our study, the overall relapse rates were 26.0% among all 527 patients over 2 years, whereas Feagan *et al*. previously reported that 41% of UC patients with oral 5-ASA relapsed [4]. The lower relapse rates in the present study may be explained by our inclusion of more patients with a longer duration of disease remission.

In a recent study, approximately half of the patients who obtained complete remission after daily treatment with 4.8 g of 5-ASA and who received maintenance therapy with 2.4 g had a relapse of the disease within 12 months [9]. These results suggest that patients with a shorter duration of disease remission who receive a lower dose of 5-ASA as maintenance therapy are at high risk of relapse. Thus, it is difficult to decide when the dose of 5-ASA can be reduced even after clinical remission has been achieved. We aimed to determine whether longer clinical remission is related to a better outcome in patients treated with 5-ASA. In our study, Fig 2 indicates that relapse rates were markedly lower after more than 2 years of remission. The results from our study indicated that reducing the dose of 5-ASA within a short duration should not considered after achieving clinical remission.

Several studies have investigated the relapse rate after clinical remission induced by a steroid. Some studies showed that the need for a steroid is related to poor prognosis in UC patients [8, 13, 14]. Khan *et al*. reported that 65% of patients whose clinical remission was induced by steroids required “re-steroid treatment” within 2 years [15]. Another study reported that the relapse rate after remission was induced by steroids was 72% in a median follow-up of 83 months among UC patients who received 5-ASA as the maintenance treatment and did not receive thiopurine [16]. These results indicate that clinical relapse is frequently observed after clinical remission has been obtained with corticosteroids. In our study, the risk of clinical relapse in the LDG patients was higher in UC patients with a history of steroid use. These data were consistent with those from another study indicating that patients with moderately active UC who had previously been treated with a corticosteroid might benefit from induction therapy with a higher dose of 5-ASA [14]. Because patients with previous use of steroids are at risk for clinical relapse, UC patients who have such a history should not be treated with a reduced dose of 5-ASA.

Thiopurines are also useful as an alternative to 5-ASA for maintenance therapy after remission has been induced by steroids. A meta-analysis found that thiopurines were more effective than placebo for the prevention of UC relapse [17]. Ardizzone et al. reported that thiopurines were significantly more effective than 5-ASA for steroid-dependent UC [18]. However, many adverse effects are associated with thiopurine use, including myelotoxicity, hepatotoxicity, pancreatitis and lymphoma. Almost half of patients who received thiopurine therapy showed adverse effects [19] during the first 12 months after receiving thiopurine. Furthermore, some patients are intolerant to thiopurine, and some do not want to use thiopurine as a maintenance therapy. Therefore, maintenance therapy with an adequate dose of 5-ASA is critical for patients who do not want to receive thiopurine, regardless of the reason.

The present study had some limitations. First, although we analysed a relatively large number of UC patients treated with oral 5-ASA alone, colonoscopy was not done in more than half of the patients. Because our study was performed for patients with clinical remission, the participants in our study didn't necessarily have to undergo annually colonoscopy in the clinical setting. However, we could confirm the endoscopic severity was associated to the prognosis and our results are consistent with many other studies demonstrating that mucosal healing is associated with prolonged disease remission. [12, 20–23]. Second, we neither used a self-administered questionnaire nor confirmed the numbers of prescribed 5-ASA tablets remaining at each visit to assess the adherence. However, we defined poor adherence to medication before the analysis of our findings. Third, our study was performed only in a Japanese population, and we did not confirm our findings in patients of other races.

## Conclusion

UC patients with a shorter duration of remission have a high risk of relapse compared to those with a long duration of remission. Therefore, the dose of 5-ASA may be reduced when the duration of disease remission is more than 2 years. In addition, for UC patients with a history of steroid use, the dose of 5-ASA should be reduced with caution because a low dose of 5-ASA increases relapse in those patients.

## Supporting information

S1 Dataset 5-ASA data. This is data in our study.
(XLSX)

## Abbreviations

5-ASA: 5-aminosalicylic acids
UC: ulcerative colitis
Alb: albumin
TC: total cholesterol
WBC: white blood cell count
Hb: hemoglobin
Plt: platelet, serum
CRP: C-reactive protein
ESR: erythrocyte sedimentation rate
MES: Mayo endoscopic score
